# TNF Is Partially Required for Cell-Death-Triggered Skin Inflammation upon Acute Loss of cFLIP

**DOI:** 10.3390/ijms21228859

**Published:** 2020-11-23

**Authors:** Maria Feoktistova, Roman Makarov, Martin Leverkus, Amir S. Yazdi, Diana Panayotova-Dimitrova

**Affiliations:** Department of Dermatology and Allergology, University Hospital RWTH Aachen, Pauwelsstraße 30, 52074 Aachen, Germany; mfeoktistova@ukaachen.de (M.F.); rmakarov2000@gmail.com (R.M.); mleverkus@ukaachen.de (M.L.); ayazdi@ukaachen.de (A.S.Y.)

**Keywords:** keratinocytes, cell death, inflammation, cFLIP, TNF, skin

## Abstract

cFLIP is required for epidermal integrity and skin inflammation silencing via protection from TNF-induced keratinocyte apoptosis. Here, we generated and analyzed cFLIP epidermal KO mice with additional TNF deficiency. Intriguingly, the ablation of TNF rescued the pathological phenotype of epidermal cFLIP KO from characteristic weight loss and increased mortality. Moreover, the lack of TNF in these animals strongly reduced and delayed the epidermal hyperkeratosis and the increased apoptosis in keratinocytes. Our data demonstrate that TNF signaling in cFLIP-deficient keratinocytes is the critical factor for the regulation of skin inflammation via modulated cytokine and chemokine expression and, thus, the attraction of immune cells. Our data suggest that autocrine TNF loop activation upon cFLIP deletion is dispensable for T cells, but is critical for neutrophil attraction. Our findings provide evidence for a negative regulatory role of cFLIP for TNF-dependent apoptosis and partially for epidermal inflammation. However, alternative signaling pathways may contribute to the development of the dramatic skin disease upon cFLIP deletion. Our data warrant future studies of the regulatory mechanism controlling the development of skin disease upon cFLIP deficiency and the role of cFLIP/TNF in a number of inflammatory skin diseases, including toxic epidermal necrolysis (TEN).

## 1. Introduction

Tumor necrosis factor (TNF) is a pleiotropic cytokine which is critical for retaining tissue homeostasis by controlling both cell death and inflammation. Several checkpoints decide if TNF will lead to cell death or survival [1]. The first checkpoint is the TNF-R1 signaling complex (complex I), which forms upon TNF-R1 activation. The signaling mediated by this complex triggers the activation of NF-κB and mitogen-activated protein kinase (MAPK) signaling, which results in the secretion of proinflammatory cytokines, including TNF [2]. Alternatively, TNF-R1 signaling can result in the formation and activation of secondary cytoplasmic complexes IIa and IIb (ripoptosome). Complexes II are formed upon interaction among RIPK1, adaptor protein FADD, caspase-8, and its protease deficient homolog cFLIP. Cleavage of caspase-8 on this platform results in the activation of apoptotic cell death. Alternatively, RIPK1 can directly interact with RIPK3, which activates necroptotic cell death. cFLIP has a critical role in the apoptosis or necroptosis decision within the ripoptosome [3].

The skin provides the first mechanical and immunological barrier protecting the body from environmental or microbial danger, and the maintenance of the skin homeostasis is of critical importance [4]. The key molecules in cell death signaling, caspase-8, FADD, and cFLIP, were shown to be essential for the maintenance of skin homeostasis [5,6,7], since loss of any of these molecules resulted in severe inflammatory skin disease. The constitutive deletion of caspase-8 or FADD in keratinocytes was demonstrated to result in necroptotic cell death and skin inflammation [5,6]. In contrast, the acute loss of cFLIP in adult skin resulted in a severe inflammatory skin disease due to massively increased apoptosis in keratinocytes [7,8]. In accordance, while the embryonic lethality of caspase-8-deficient animals is rescued by concomitant ablation of RIPK3, cFLIP-deficient animals are only able to survive when both RIPK3 and FADD are additionally deleted [9]. The apoptotic cell death in cFLIP-deficient keratinocytes is dependent on the autocrine TNF loop triggered by deletion of cFLIP [7]. The inhibition of autocrine TNF signaling by TNF-R2-Fc results in partial protection of cFLIP-deficient keratinocytes from cell death and an ameliorated pathological skin phenotype [7]. As a cFLIP-deficient skin strongly resembles the typical skin morphology of patients with toxic epidermal necrolysis (TEN), we previously suggested that cFLIP deficiency is a possible prerequisite for the fulminant cell death in the skin of TEN patients [7,10]. In accordance with these data, a study reported that an early inhibition of the TNF signaling by TNF-R-Fc resulted in complete healing of 10 patients with TEN [11], confirming the critical role of TNF-dependent cell death in this pathology.

In this work, we generated and analyzed a double-knockout mouse model lacking TNF completely and cFLIP exclusively in keratinocytes. With this new genetic mouse model, we can confirm that the inflammatory skin disease that results from the epidermal deletion of cFLIP is strongly but not exclusively dependent on TNF. Additional, yet unknown signaling pathways beyond TNF signaling may play a role in the cell death and inflammatory pathogenicity in cFLIP-deficient skin.

## 2. Results

### 2.1. TNF Deficiency Delays the Development of Inflammatory Skin Disease and Protects from Weight Loss and Mortality upon cFLIP Deletion

We could previously show the role of the TNF autocrine loop for the cell death induced in cFLIP-deficient keratinocytes in vivo [7]. In our previous experiments, we used a soluble TNF receptor 2 fusion protein (recombinant TNF-R2-Fc) for inhibition of TNF-dependent signaling in the skin of cFLIP epidermal KO mice [7]. In the present study, we aimed to test whether genetic ablation of TNF could completely prevent the strong skin phenotype induced by cFLIP ablation. To address this aim, we generated mice with temporally and spatially controlled deletion of cFLIP in keratinocytes and complete lack of TNF by crossing cFLIP^fl/fl^-K14CreER^tam^ mice with TNF^−/−^ mice.

**Figure 1:**
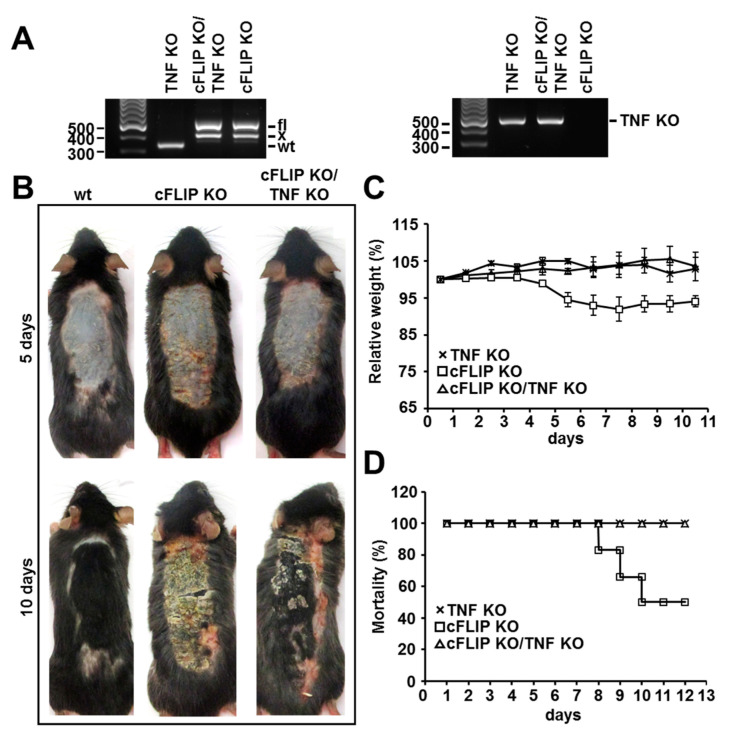
TNF promotes inflammatory skin disease, increased weight loss, and mortality in epidermal cFLIP KO. (**A**) Detection of tamoxifen-induced excision of the cFLIP allele (x) and TNF deficiency in the epidermis. DNA was isolated from the epidermis of TNF KO, cFLIP KO, and cFLIP KO/TNF KO mice and analyzed using PCR. fl, floxed allele; WT, wild-type allele; x, excised allele. (**B**) Macroscopic phenotype of control, cFLIP KO, and cFLIP KO/TNF KO animals at 5 and 10 days. Representative photographs of mice (6–10 mice per group) are shown. (**C**) Relative weight and (**D**) survival analysis (*n* = 6) of TNF KO, cFLIP KO, and cFLIP KO/TNF KO mice after 5 days of tamoxifen application.

To achieve exclusively epidermal cFLIP deletion in adult animals, cFLIP^fl/fl^-K14CreER^tam^ (cFLIP KO), TNF^−/−^cFLIP^fl/fl^-K14CreER^tam^ (cFLIP KO/TNF KO), and TNF^−/−^ (TNF KO) animals were epilated, and tamoxifen was applied for 3–5 days on the epilated back skin. Successful deletion of cFLIP fl/fl alleles upon tamoxifen application, as well as TNF deficiency, was confirmed by PCR (Figure 1A). The epidermal ablation of cFLIP in TNF-deficient animals resulted in a significant amelioration of clinically evident skin inflammation compared to cFLIP animals not lacking TNF (Figure 1B) 5 days after the first tamoxifen application. Loss of TNF delayed the appearance of the typical macroscopic skin alterations in cFLIP-deficient skin, such as reddening, crusting, and skin thickening, but was unable to completely rescue the skin phenotype. However, the skin of animals lacking TNF recovered faster compared with the TNF-expressing cFLIP epidermal KO animals (Figure 1B). Importantly, the severe weight loss and the mortality of mice with acute epidermal cFLIP deletion was completely rescued by TNF deficiency (Figure 1C,D). Together, these observations suggest that cFLIP-controlled systemic symptoms such as weight loss and mortality are TNF-dependent, while the skin reaction only partially depends on TNF.

### 2.2. TNF Deficiency Protects cFLIP-Deficient Epidermis from Hyperproliferation, Dysregulated Differentiation, and Apoptosis

**Figure 2:**
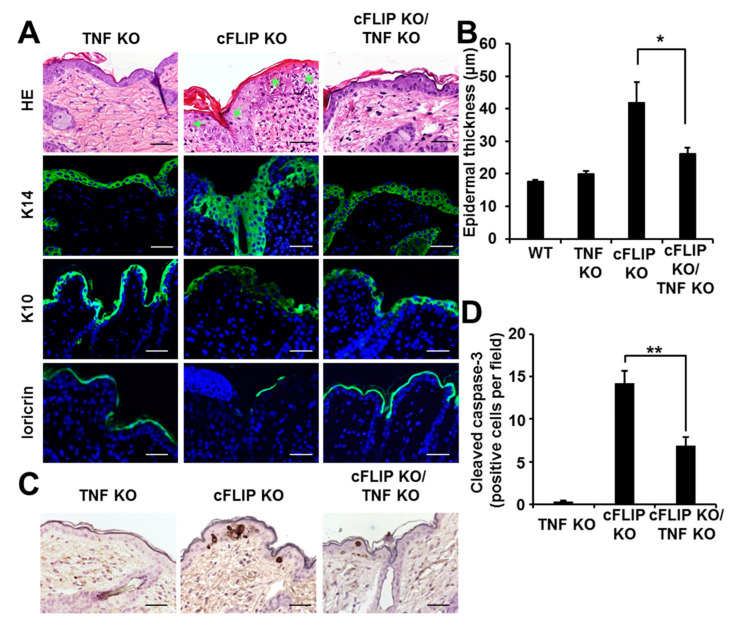
TNF signaling is required for epidermal hyperplasia upon acute loss of cFLIP and is critical for epidermal apoptosis in the absence of cFLIP. (**A**). Skin sections at T72H from TNF KO, cFLIP KO, and cFLIP KO/TNF KO animals stained with hematoxylin and eosin staining (H&E), keratin 14, keratin 10, and loricrin. Arrows: pyknotic nuclei; stars: intraepidermal pustules. Scale bars represent 50 µm. Blue: DAPI; green: K14 or K10 staining, respectively. (**B**). Epidermal thickness of skin isolated at T72H from TNF KO, cFLIP KO, and cFLIP/TNF KO mice, compared to skin isolated from wild-type mice (5–6 animals per group). (**C**). Skin section collected at T72H and stained with H&E along with an antibody against cleaved caspase-3 (brown). Scale bars represent 50 µm. (**D**). Quantification of the cleaved caspase-3-positive cells per microscopic field; * *p* < 0.05; ** *p* < 0.01.

We next examined the early skin phenotype of cFLIP KO/TNF KO mice ex vivo. The immunohistological analysis of skin isolated 72 h after first tamoxifen application (T72H) demonstrated that TNF deficiency partially protected cFLIP-deficient epidermis from the characteristic acanthosis and spongiosis (Figure 2A). The typical acanthotic thickening of the epidermis upon cFLIP deletion was significantly reduced by the absence of TNF. Keratin 14 (K14), keratin 10 (K10), and loricrin stainings demonstrated impaired and dysregulated keratinocyte differentiation in cFLIP-deficient mice whenever TNF was deleted (Figure 2A,B). We previously showed that cFLIP deficiency in epidermis results in fulminant apoptotic cell death and loss of the epidermal layer [7]. In the present study, we addressed whether genetic TNF ablation was sufficient for the complete blockage of cFLIP-controlled apoptosis. Immunohistochemical staining of skin isolated at T72H for active caspase-3 demonstrated a significant reduction in apoptotic keratinocytes in cFLIP-deficient epidermis when TNF was deleted (Figure 2C,D). In contrast, in the cFLIP-deficient animals with additional deletion of RIPK3, active caspase-3 remained unaltered (data not shown). Thus, we could confirm that TNF is the critical factor for apoptosis in keratinocytes in the absence of cFLIP.

### 2.3. The Inflammatory Response in the Skin of Epidermal cFLIP KO Is Partially Dependent on TNF

**Figure 3:**
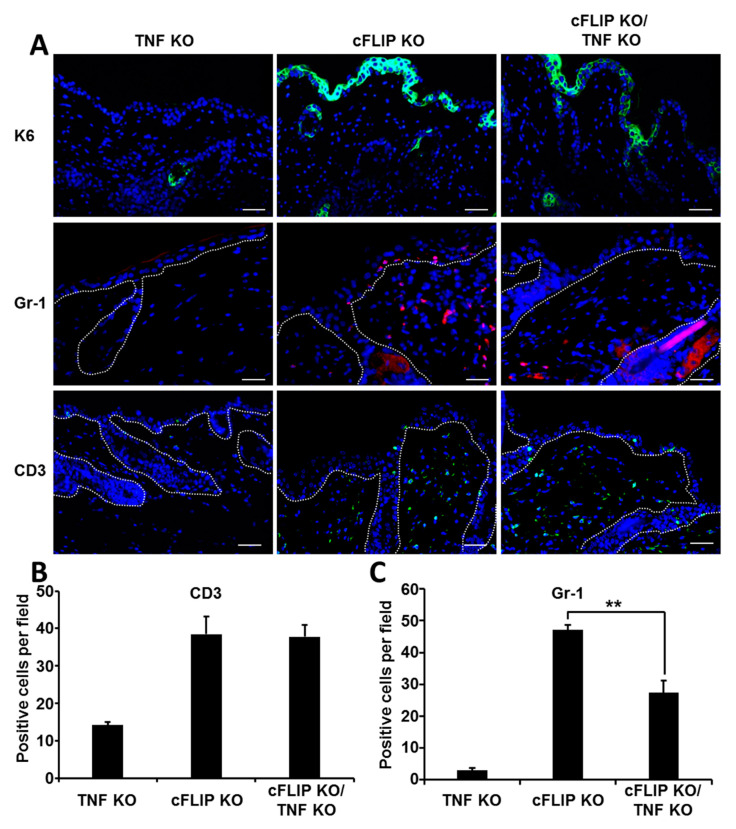
TNF signaling is required for the rapid dermal influx of granulocytes, but not for CD3^+^ T cells in cFLIP-deficient epidermis. (**A**). Skin section at T72H isolated from TNF KO, cFLIP KO, and cFLIP KO/TNF KO animals stained for Gr-1, CD3, and keratin 6. Blue: DAPI; green: K6, or CD3^+^T, respectively; red: Gr-1 staining. White dashed line: boundary between epidermis and dermis. Scale bars correspond to 50 µm. Quantification of (**B**) CD3- and (**C**) Gr-1-positive cells per microscopic field; ** *p* < 0.01.

We next investigated the role of TNF for the inflammatory response in the skin of epidermal cFLIP-deficient animals. Staining for keratin 6 (K6), known to be expressed in the epidermal layer only during inflammatory disease, demonstrated reduced expression in the epidermis of cFLIP KO/TNF KO mice (Figure 3A). Immunohistological staining demonstrated infiltration of CD3^+^ T cells at T72H in both cFLIP KO and cFLIP KO/TNF KO animals (Figure 3A). Quantification of the infiltrated CD3^+^ cells revealed no TNF dependency on the number of infiltrating T cells in the skin of cFLIP epidermal KO animals (Figure 3B). Interestingly, in contrast to CD3^+^ cells, the number of infiltrating Gr-1-positive cells was significantly reduced in the skin of cFLIP KO/TNF KO animals (Figure 3A,C).

**Figure 4:**
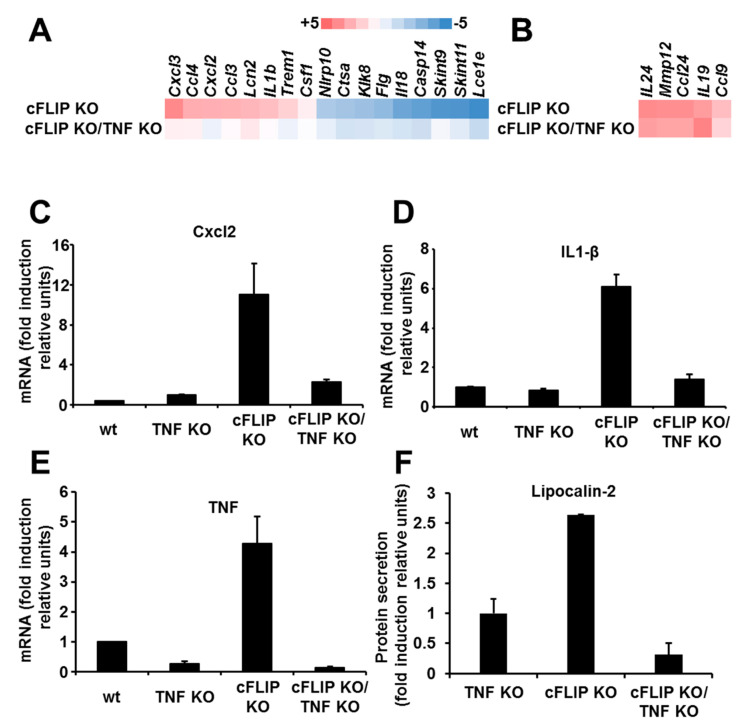
TNF-dependent modulation of gene expression in cFLIP-deficient epidermis. (**A**,**B**) Heat maps of inflammatory genes related to (**A**) TNF-dependent and (**B**) TNF-independent genes in epidermis isolated from cFLIP KO and cFLIP KO/TNF KO mice compared to TNF KO mice. The array experiment was performed with messenger RNAs (mRNAs) pooled from 3–6 animals per genotype. (**C**–**E**) mRNA expression of (C) Cxcl2, (D) Il-1β, and (**E**) TNF in epidermis isolated from control, TNF KO, cFLIP KO, and cFLIP KO/TNF KO mice. Error bars represent the standard error of the mean (SEM) of two independent experiments. (**F**). Lipocalin-2 secretion from immortalized keratinocytes isolated from TNF KO, cFLIP KO, and cFLIP KO/TNF KO mice. Immortalized keratinocytes were pooled from three independent experiments.

To gain further insights into the role of TNF for the inflammatory response in epidermal cFLIP KO skin, we next performed gene expression profiling to compare epidermal messenger RNA (mRNA) isolated from TNF KO, cFLIP KO, and cFLIP KO/TNF KO animals at T72H. Focusing on inflammation-related genes involved in the attraction of inflammatory cells, we detected a group of genes known to be related to modulation of T cell activity and attraction [12,13], which were upregulated in the cFLIP-deficient epidermis independently of TNF (Figure 4B). These data are in line with the unchanged number of infiltrating CD3-positive cells in the skin of both cFLIP KO and cFLIP KO/TNF KO animals. In contrast, several genes involved in the attraction of neutrophils, such as Cxcl2, Lcn-2, and Ccl4, demonstrated TNF-dependent upregulation in cFLIP-deficient epidermis (Figure 4A), corresponding to the decreased number of Gr-1-positive granulocytes. Moreover, we detected increased secretion of Lipocalin-2 from immortalized keratinocytes upon cFLIP deletion in a TNF-dependent manner (Figure 4F). Additional quantitative real-time PCR analysis of epidermal mRNA isolated from cFLIP KO and cFLIP KO/TNF KO animals demonstrated a lack of upregulation of the proinflammatory genes IL-1β and CXCL2 in epidermis of cFLIP KO/TNF KO, suggesting that the regulation of both inflammatory genes in cFLIP-deficient keratinocytes is TNF-mediated (Figure 4C–E). Additionally, the gene expression profile demonstrated a significant TNF-dependent downregulation of genes related to the epidermal barrier complex, such as Kallikrein-related peptidase-8 (Klk8), Filaggrin (Flg), Il-18, and caspase-14 (Casp14) [14,15,16,17] (Figure 4A). This might explain the ameliorated skin phenotype in cFLIP KO/TNF KO.

## 3. Discussion

cFLIP, FADD, and caspase-8 are key molecules controlling programmed cell death. Our previous studies demonstrated the crucial regulatory function of cFLIP isoforms for the formation and regulation of the intracellular signaling platform called the ripoptosome. This platform is capable of mediating both apoptotic and necroptotic signaling pathways [3]. FADD or caspase-8 deficiency in skin results in spontaneous necrosis of keratinocytes in vivo, causing an inflammatory skin disease [5,6].

**Figure 5:**
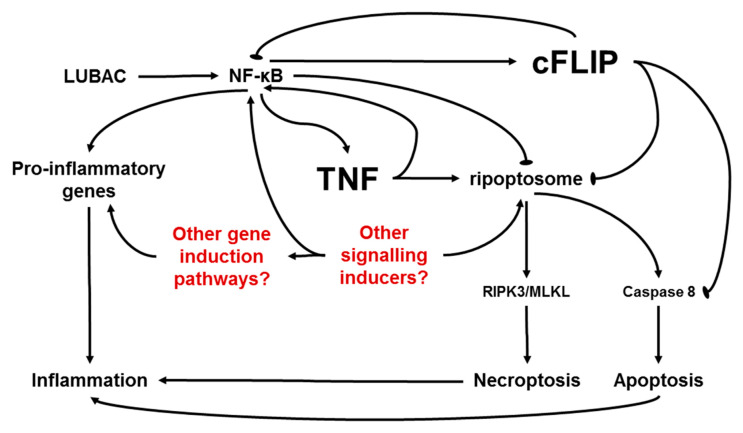
Suggested mechanism of NF-κB-mediated cell death and inflammatory signaling in keratinocytes with dysregulated homeostasis. The LUBAC is required for NF-κB activation which promotes expression of target genes, including proinflammatory genes, cFLIP (NF-κB inhibitor), and TNF (NF-κB inducer). TNF-induced signaling can lead either to inflammation or to cell death via the ripoptosome. The ripoptosome is able to direct the cell to either necroptosis (through RIPK3/MLKL activation) or apoptosis (though caspase cascade activation). cFLIP can block the apoptotic function of the ripoptosome, and NF-κB can entirely block the formation of the ripoptosome. Possible additional factors involved in the suggested mechanism are marked in red.

In a previous study, we clarified the role of cFLIP for skin homeostasis and integrity in vivo. We demonstrated that the fulminant cell death in cFLIP-deficient keratinocytes in vivo and in vitro is exclusively apoptotic and can be inhibited by TNF-neutralizing antibodies [7]. Both in vitro and in vivo, cFLIP-deficient keratinocytes require the activated TNF-mediated autocrine loop in order to initiate TNF-mediated apoptosis. Here, we confirmed that TNF is the critical factor for epidermal apoptosis in the cFLIP-deficient epidermis. The protective role of cFLIP for apoptotic cell death was greatly dependent on TNF, as the rapid appearance of active caspase-3 in the epidermal keratinocytes was strongly inhibited in the skin of TNF-deficient cFLIP epidermal KO animals. cFLIP was suggested to be a critical factor for the inhibition of TNF complex II [18], as well as the ripoptosome [3]. In the present study, we used a new genetic model with TNF deficiency and inducible deletion of the epidermal cFLIP. Upon acute deletion of cFLIP in adult skin, homeostasis was disturbed, the animals lost weight, and some of the animals could not recover and died. We now show that both increased weight loss and partial mortality were promoted by TNF signaling. Interestingly, no complete rescue of the clinical skin phenotype was identified. Epidermal cFLIP KO mice lacking TNF also developed an ameliorated inflammatory skin macroscopic phenotype, which was delayed in comparison to the TNF-expressing animals. These results are in line with the data obtained from mice lacking caspase-8 or FADD in the skin, where the deletion of TNF or TNF-R1 also delayed the skin phenotype [5,6]. Severe dysregulation of keratinocyte homeostasis was also demonstrated in a genetic mice model with specific keratinocyte deletion of HOIP and HOIL, components of the linear ubiquitin chain assembly complex (LUBAC) [19,20]. HOIL and HOIP epidermal KO mice demonstrated fulminant keratinocyte cell death and lethal dermatitis. Despite the probably different mechanisms driving cell death induction versus inflammatory skin disease (Figure 5) in these mouse models, TNF signaling was shown to be only partially involved in this process.

On the basis of the present data and published reports regarding dysregulation of the keratinocyte homeostasis resulting in cell death and inflammation, we postulate the following scenario: NF-κB activation promotes the expression of numerous target genes, including proinflammatory genes, cFLIP (NF-κB inhibitor), and TNF (NF-κB inducer). The LUBAC is critically required to activate NF-κB signaling; therefore, absence of the LUBAC may lead to ripoptosome formation and cell death. cFLIP deficiency results in blocking of NF-κB [21], promotion of ripoptosome formation, caspase-8 activation, and apoptosis [3]. Abrogation of TNF signaling results in ameliorated and delayed inflammatory skin disease. TNF signaling is able to promote both inflammation and cell death most probably via the ripoptosome. Since, in both cFLIP KO/TNF KO and LUBAC component KO animals, cell death and inflammation were not completely blocked, we hypothesize that there are other inflammation/cell death inducers, which are capable of inducing either NF-κB or other proinflammatory signaling pathways. As the keratinocytes are strategically the first line of defense against environmental factors, it is not surprising that they also play an important role in the regulation of the inflammatory response in the skin [4]. A number of proinflammatory signaling pathways can be activated in the epidermis of patients suffering from various inflammatory skin disease [22,23,24]. Moreover, it was recently demonstrated that specific CARD14 signaling in keratinocytes is responsible for psoriasiform skin inflammation in mouse models, independently of the adaptive immune system [25]. Our data highlight that TNF signaling in cFLIP-deficient keratinocytes is the critical factor for the regulation of skin inflammatory responses via modulated cytokine and chemokine expression and, therefore, the attraction of immune cells. Our data suggest that autocrine TNF loop activation upon cFLIP deletion is dispensable for the expression of cytokines responsible for T-cell attraction, but is critical for the increased expression of proteins attracting neutrophils. We detected a TNF-dependent upregulation of several known chemoattractants for neutrophils, such as Cxcl2 and Lcn-2. Lcn-2 is upregulated in psoriatic skin both in human and in mice models, and it appears to have a pathogenic role in psoriasis [26,27]. Lcn-2 was not only upregulated in circulating neutrophils in psoriatic patients, but also in activated keratinocytes [27]. Our TNF/cFLIP epidermal KO mice model reacted with a TNF-dependent increased Lcn-2 expression in epidermis in line with a massive infiltration of neutrophils. Importantly, we showed that immortalized keratinocytes secreted increased amounts of Lipocalin-2 upon cFLIP deletion in a TNF-dependent manner. Epidermal dysfunction and keratinocyte injury were suggested to be at least some of the causes inducing keratinocytes to secrete Lcn-2 in psoriasis [27]. Consistent with this, keratinocyte-specific ablation of cFLIP resulted in dysregulated cellular homeostasis, which promoted the expression of proinflammatory genes related to neutrophil attraction, including Lcn-2. Additionally, we detected TNF-dependent changes in gene expression related to the epidermal barrier. This can explain the ameliorated macroscopic skin manifestation in the cFLIP epidermal KO animals lacking TNF.

Previous reports using genetic models indicated that dysregulation of cell death and TNF signaling in keratinocytes is a potent inducer of inflammatory skin disease [5,6,7,19,20,28]. In accordance, TNF is not the single driver of cell-death-induced inflammation in the skin of cFLIP epidermal KO animals. Alternatively, TNF-independent signaling pathways must be activated upon cFLIP deletion as well, which still has to be determined. For example, it would be interesting to analyze the role of TNF-like weak inducer of apoptosis (TWEAK) signaling, since this pathway was shown to contribute to the control of skin homeostasis and inflammation and have a role in the development of the inflammatory skin disease psoriasis [29,30,31]. Taken together, our findings provide insights into the regulation of the epidermal cell death and inflammation and warrant future analyses of the impact cFLIP and TNF in a number of inflammatory skin pathologies including psoriasis and toxic epidermal necrolysis.

## 4. Materials and Methods

### 4.1. Mice

To generate cFLIP^fl/fl^ K14CreER^tam^ TNF^−/−^ (cFLIP KO/TNF KO) mice, cFLIP^fl/fl^ K14CreER^tam^ (cFLIP KO) mice [7] were crossed with TNF KO mice. cFLIP deletion was induced by topical application of tamoxifen (25 mg/mL) (Sigma, St. Louis, MO, USA) on restricted, epilated back skin. Tamoxifen was applied for 3–5 days. All animals in this study were treated in full compliance with the guidelines for animal care approved by the institutional German Animal Care Committee (LANUV, Recklinghausen, Germany; Az.: 84-02.04.2016.A003).

### 4.2. Genotyping Multiplex PCR

DNA was isolated from the epidermis using a standard DNA isolation procedure. The presence of the wild-type, floxed, or Cre-excised (deleted) cFLIP allele was confirmed by multiplex PCR. Three pairs of primers were used for simultaneous detection of wild-type (wt), cFLIP floxed (fl), and excised (x) allele as outlined. TNF deletion was confirmed by conventional PCR. Primer sequences are available upon request.

### 4.3. Histology and Immunohistochemistry

Firstly, 72 h after the first tamoxifen application, skin was isolated, formalin-fixed, and paraffin-embedded. The following primary antibodies were used for fluorescence microscopy: K14, K10, loricrin (Covance, Princeton, NJ, USA), and cleaved caspase-3 (Asp175; Cell Signaling Technology, Danvers, MA, USA). Secondary biotin-conjugated antibodies were from Vector Laboratories (Burlingame, CA, USA), i.e., Cy-2/Cy-3-conjugated streptavidin. Image processing was applied identically for all samples and controls. Quantification of or immunofluorescence labeling of histological slides was performed semiquantitatively by three independent investigators blinded to the treatment of the respective mouse in at least 20–25 fields of each sample.

### 4.4. Primary Keratinocyte Isolation and Immortalization

PKs were isolated from newborn complete skin or adult tail skin and immortalized using spontaneous immortalization CnT-PR medium (CELLnTEC, Bern, Switzerland). Immortalized keratinocytes were transduced with an LV construct expressing Cre-Recombinase or control LV.

### 4.5. Lentiviral Expression of Cre in PKs and Immunoblotting

The pFU Cre SV40 puro W construct was kindly provided by John Silke (WEHI, Melbourne, Australia). Lentivirus-expressing Cre-recombinase was generated in HEK cells.

### 4.6. Statistical Analysis

The standard error of mean (SEM) was determined for three independent experiments performed with immortalized keratinocytes pooled from 3–5 newborn animals. Student’s *t*-test was used for statistical data analysis. Significance is stated whenever *p* was <0.05.

### 4.7. RNA Isolation and Real-Time qPCR

RNA isolation from epidermis or cultured PKs was performed with RNeasy Kit (Qiagen, Venlo, The Netherlands; Hilden, Germany). For complementary DNA (cDNA) synthesis, SuperScript II Reverse Transcriptase (Invitrogen, Waltham, MA, USA) was used with a mixture of random nanomers and oligo dT primers in a ratio 10:1. RT-qPCR analysis was performed using KAPA SYBR Fast qPCR (Peqlab, Erlangen, Germany) in the Abi 7300 (Thermo Fischer Scientific, Waltham, MA, USA) real-time thermal cycler. Equal cycling conditions were used to amplify genes of interest and reference gene products. Mean values were calculated using data obtained from three independent experiments. Normalization of each experiment was performed to β-actin expression. Primer sequences and details for qPCR cycling conditions are available upon request.

### 4.8. Affymetrix GeneChip Oligoarray Analysis

mRNA from was isolated from epidermis of 3–6 animals per group and pooled. The Affymetrix GeneChip Mouse Gene 2.0 ST Array (Thermo Fisher Scientific, Waltham, MA, USA) was used according to the manufacturer’s instructions. A Custom CDF V. 22 with ENTREZ-based gene definitions was used to annotate the arrays [32]. Quantile normalization and robust multiarray analysis background correction were used for normalization of fluorescence intensity. An analysis of variance was performed to identify differentially expressed genes using the commercial software package SAS JMP Genomics, V. 7 (SAS Institute, Cary, NC, USA). The level of significance was a 0.05 false positive rate with false discovery rate correction (GEO accession number: GSE161649).
